# Supplementary material for: Mediating reconciliation with God: Exploring divine forgiveness experiences during confession among Catholic priests from four Spanish-speaking countries
Source: PLoS One. 2026 May 11;21(5):e0347608. doi: 10.1371/journal.pone.0347608 (PMC13160297; doi:10.1371/journal.pone.0347608)
Supplement: S1 Table — The Consolidated Criteria for Reporting Qualitative Research (COREQ) is a 32-item checklist designed to improve transparency and completeness in reporting qualitative studies based on interviews and focus groups. Although the information corresponding to these criteria is integrated throughout the manuscript in the relevant methodological and analytic sections, all COREQ items are compiled here in a single supplementary table to provide a clear and comprehensive overview of how each criterion was addressed in the design, conduct, analysis, and reporting of the present study. (DOCX) [file pone.0347608.s001.docx]

**Table S1. COREQ 32-Item Checklist for the present study**

| **Domain / Item** | **COREQ Question** | **Response for This Study** |
| --- | --- | --- |
| **DOMAIN 1: Research Team and Reflexivity** | | |
| **1. Interviewer/ facilitator** | Which author conducted the interviews? | Interviews were conducted by [omitted] and an external male collaborator from a [omitted] institution, both familiar with qualitative interviewing. |
| **2. Credentials** | What were the researcher’s credentials? | All authors hold a PhD. |
| **3. Occupation** | What was their occupation at the time of the study? | All authors were full-time academic researchers at universities. |
| **4. Gender** | Was the researcher male or female? | Both genders were represented in the research team. The interviews were conducted by two male researchers, whereas data analysis was led by two female researchers and triangulated with an additional male researcher. |
| **5. Experience and training** | What experience or training did the researcher have? | One expert in qualitative research, four with prior experience, and one conducting qualitative interviewing for the first time. |
| **6. Relationship established** | Was a relationship established prior to the study? | Yes, with the two pilot participants and with four of the interviewed priests. |
| **7. Participant knowledge of the interviewer** | What did participants know about the researcher? | They knew the study explored psychological aspects of confession; final aims were not disclosed to avoid influencing responses. |
| **8. Interviewer characteristics** | Bias, assumptions, interests in topic? | Researcher reflexivity and positionality were explicitly discussed in the manuscript. |
| **DOMAIN 2: Study Design** | | |
| **9. Methodological orientation** | What methodological orientation was used? | A theory-informed framework analysis combining inductive coding and matrix-based synthesis. |
| **10. Sampling** | How were participants selected? | Purposive and snowball sampling using email, phone, and WhatsApp in Spanish. Recruitment followed predefined diversity criteria. |
| **11. Method of approach** | How were participants approached? | Email, phone calls, and WhatsApp messages. |
| **12. Sample size** | How many participants? | Ten priests. Saturation occurred at interview 8; two additional interviews confirmed it. |
| **13. Non- participation** | How many refused or dropped out? | Three priests declined due to lack of time. |
| **14. Setting of data collection** | Where was data collected? | In-person at the university or online via video call from parishes or community residences. |
| **15. Presence of non-participants** | Anyone else present? | No. |
| **16. Description of sample** | Key characteristics? | Ten Catholic priests: diocesan and religious (Jesuits, Franciscans, Opus Dei); native Spanish speakers; active confessors; ≥15 years ordained. |
| **17. Interview guide** | Were questions available? Piloted? | Yes. The interview guide is publicly available; two pilot interviews were conducted. |
| **18. Repeat interviews** | Were repeat interviews conducted? | No. |
| **19. Recording** | Audio/visual recording used? | Audio recordings were used. |
| **20. Field notes** | Field notes made? | Yes. Pre- and post-interview notes documented context, complications (none), and early analytic memos. |
| **21. Duration** | Duration of interviews? | 60–99 minutes (M = 74). |
| **22. Data saturation** | Was saturation discussed? | Yes. Saturation was reached at the eighth interview; final two confirmed it. |
| **23. Transcripts returned** | Were transcripts returned? | Only pilot transcripts were returned for accuracy checking. |
| **DOMAIN 3: Analysis and Findings** | | |
| **24. Number of data coders** | How many coders? | Two researchers coded independently; discrepancies were resolved with a third. |
| **25. Description of coding tree** | Was a coding tree described? | Yes. A three-level coding structure (specific codes, broader categories, overarching themes) is provided in Supplementary Table S1. |
| **26. Derivation of themes** | Themes pre-set or emergent? | Themes emerged inductively but were interpreted through a theory-informed lens (Cook & Cowden, 2025). |
| **27. Software** | What software was used? | Taguette. |
| **28. Participant checking** | Did participants give feedback on findings? | Pilot participants provided feedback. Additionally, findings were presented to a group of priests to assess thematic resonance. |
| **29. Quotations presented** | Were quotations included? | Yes. Quotations are presented in the manuscript and in a supplementary matrix by code. |
| **30. Data–findings consistency** | Were findings consistent with data? | Yes. Consistency was ensured through triangulation, iterative refinement, and credibility procedures. |
| **31. Clarity of major themes** | Were major themes clearly presented? | Yes, they are clearly articulated and discussed in the Results section. |
| **32. Clarity of minor themes** | Were minor themes / variations described? | Yes. Minor themes, divergent cases, and exceptions were explicitly reported and discussed. |

*Note.* The Consolidated Criteria for Reporting Qualitative Research (COREQ) is a 32-item checklist designed to improve transparency and completeness in reporting qualitative studies based on interviews and focus groups. Although the information corresponding to these criteria is integrated throughout the manuscript in the relevant methodological and analytic sections, all COREQ items are compiled here in a single supplementary table to provide a clear and comprehensive overview of how each criterion was addressed in the design, conduct, analysis, and reporting of the present study.
