# Supplementary material for: Mediating reconciliation with God: Exploring divine forgiveness experiences during confession among Catholic priests from four Spanish-speaking countries
Source: PLoS One. 2026 May 11;21(5):e0347608. doi: 10.1371/journal.pone.0347608 (PMC13160297; doi:10.1371/journal.pone.0347608)
Supplement: S2 Table — Codes were initially informed by the Reconciliation with God framework (Cook & Cowden, 2025) and then expanded through inductive analysis beyond this framework. Coding proceeded bottom-up in three hierarchical levels: Level 3 (most specific), Level 2 (broader), and Level 1 (most general). English labels are shown with their original Spanish terms in parentheses (e.g., Repentance (Arrepentimiento)). Codes were first developed at Level 3, then clustered into broader Level 2 and Level 1 codes, which were subsequently grouped into categories, subthemes, and overarching themes. The complete coding matrix is provided in the main manuscript as a referenced supplementary file. (DOCX) [file pone.0347608.s002.docx]

**Table S2. Themes, subthemes, and associated codes (English labels with Spanish terms in parentheses)**

| **Subtheme** | **Codes (English · Spanish)** |
| --- | --- |
| **1) Living Confession: The Relational Fabric of Experience** | |
| **The Vulnerable Self in Confession** | **L1:** Penitent’s Experiences & Factors (Experiencias y factores del penitente). **L2:** Penitent’s Personal Experience (Experiencia personal penitente); Spiritual Attributes (Atributos espirituales); Penitent’s Personality (Personalidad del penitente). **L3:** Repentance (Arrepentimiento); Sorrow for sins (Dolor de los pecados); Predisposition (Predisposición); Penitent virtues (Virtudes penitente); Guilt (Culpa); Shame (Vergüenza). |
| **Personal Sin as Wound and Catalyst** | **L1:** Penitent’s Experiences & Factors. **L2:** Type of Sin (Tipo pecado). **L3:** Wound (Herida); Suffering (Sufrimiento); Conversion (Conversión); Forgiveness from others (Perdón de otros). |
| **God’s Presence and Grace** | **L1:** DFP Dimensions (Dimensiones del DFP). **L2:** Cognitive Dimension of DF (Dimensión cognitiva DF); Emotional Dimension of DF (Dimensión emocional DF). **L3:** Grace (Gracia); Experience of God (Experiencia de Dios); Image of God (Imagen Dios); Freedom (Libertad). |
| **Other People and Supportive Bonds (incl. spiritual direction)** | **L1:** Context (Relationship) (Contexto (relación)); Penitent–Confessor Relationship (Relación penitente confesor). **L2:** Spiritual direction (Dirección espiritual); Community experience (Experiencia de comunidad). **L3:** Relationship (Relación); Experience with priests (Experiencia con sacerdotes). |
| **The Confessor as the Face of God and Humanity** | **L1:** Confessor’s Experiences & Factors (Experiencias y factores del confesor); Penitent–Confessor Relationship. **L2:** Confessor factors (Factores del confesor); Actions & Avoidances (Acciones y evitaciones); Confessor’s Personal Experience (Experiencia personal del confesor). **L3:** Warm welcome (Acogimiento); Hurry (Prisa); Mediation (Mediación); Intermediary (Intermediario); Absolution (Absolución); Ministry (Ministerio). |
| **2) Dynamic Movements of Reconciliation** | |
| **Disposition and Preparation** | **L1:** Penitent’s Experiences & Factors. **L2:** Spiritual Attributes; Penitent actions (Acciones penitente). **L3:** Recollection—prayer (Recogimiento (rezar)); Predisposition; Control (Control); Confession frequency/habit (Frecuencia/hábito confesión). |
| **Repentance** | **L1:** Penitent’s Experiences & Factors. **L2:** Spiritual Attributes. **L3:** Repentance; Sorrow for sins; Conversion. |
| **Verbal Confession** | **L1:** Penitent–Confessor Relationship. **L2:** Actions & Avoidances. **L3:** Confession (Confesión); Human side (parte humana); Relationship with God (relación con Dios). |
| **Confessor’s Mediation: Accompaniment and Acceptance** | **L1:** Confessor’s Experiences & Factors. **L2:** Confessor factors. **L3:** Mediation; Intermediary; Warm welcome. |
| **Penance: Reflection and Integration** | **L1:** Penitent’s Experiences & Factors. **L2:** Actions & Avoidances. **L3:** Penance (Penitencia); Penitent actions. |
| **Absolution: Grace, Peace, and a New Beginning** | **L1:** DFP Dimensions. **L2:** Reconciliation (Reconciliation). **L3:** Absolution; Grace; Freedom. |
| **Culmination: Reconciliation and Renewal** | **L1:** DFP Dimensions; Relation to Self-Forgiveness (Relación SF). **L2:** Reconciliation; Self-Forgiveness Perception (Percepción del perdón propio). **L3:** Freedom; Flourishing (Florecimiento); Well-being (Bienestar). |
| **3) Perceived Barriers and Facilitators** | |
| **Barriers — Personal (guilt, self-image, scrupulosity, shame)** | **L1:** Relation to Mental Health (Relación con salud mental); Penitent’s Experiences & Factors. **L2:** Psychological Difficulties (Dificultades psicológicas). **L3:** Scruples (Escrúpulos); Guilt; Shame; Psychological help (Ayuda psicológica). |
| **Barriers — Relational, Ritual, and Cultural** | **L1:** Context (Relationship); Penitent–Confessor Relationship. **L2:** Relationship; Community experience; Culture/Tradition (Cultura/Tradición); Secularization (Secularización). **L3:** Hurry; Experience with priests; Event (Evento). |
| **Facilitators — Personal** | **L1:** Penitent’s Experiences & Factors. **L2:** Spiritual Attributes. Penitent virtues; Recollection—prayer; Conversion. |
| **Facilitators — Relational/Liturgical** | **L1:** Penitent–Confessor Relationship; Context (Relationship). **L2:** Spiritual direction; Community experience; Relationship. **L3:** Warm welcome; Mediation; Event. |
| **4) Cognition and Emotion in the Experience of DF** | |
| **Cognitive/ Doctrinal Dimension** | **L1:** DFP Dimensions. **L2:** Cognitive Dimension of DF. **L3:** Image of God; Freedom; Grace. |
| **Emotional/ Experiential Dimension** | **L1:** DFP Dimensions. **L2:** Emotional Dimension of DF. **L3:** Experience of God; Grace; Self-Forgiveness Perception. |
| **5) Integrative Axis: The Relational Fabric and the Dynamism of DF** | |
| **Interrelation of Axes** | **L1:** Context (Relationship); Penitent–Confessor Relationship. **L2:** Relationship. **L3:** Mediation; Intermediary; Human side; Relationship with God. |
| **Dynamic Sequence (Call & Response)** | **L1:** Penitent’s Experiences & Factors; DFP Dimensions. **L2:** Penitent actions; Actions & Avoidances. **L3:** Penitent actions; Confession frequency/habit; Conversion. |
| **Image of God & Self-Image** | **L1:** DFP Dimensions; Penitent’s Experiences & Factors. **L2:** Cognitive Dimension of DF. **L3:** Image of God; Wound. |
| **Confessor’s Climate (welcoming vs. distant)** | **L1:** Confessor’s Experiences & Factors; Penitent–Confessor Relationship. **L2:** Confessor factors. **L3:** Warm welcome; Hurry; Ministry. |
| **Forms of Verification of DF (absolution words vs. felt experience)** | **L1:** DFP Dimensions. **L2:** Cognitive Dimension of DF; Emotional Dimension of DF. **L3:** Absolution; Experience of God; Confession. |
| *Note.* Codes were initially informed by the *Reconciliation with God* framework (Cook & Cowden, 2025) and then expanded through inductive analysis beyond this framework. Coding proceeded bottom-up in three hierarchical levels: Level 3 (most specific), Level 2 (broader), and Level 1 (most general). English labels are shown with their original Spanish terms in parentheses (e.g., *Repentance* (Arrepentimiento)). Codes were first developed at Level 3, then clustered into broader Level 2 and Level 1 codes, which were subsequently grouped into categories, subthemes, and overarching themes. The complete coding matrix is provided in the main manuscript as a referenced supplementary file. | |
