# Supplementary material for: Mediating reconciliation with God: Exploring divine forgiveness experiences during confession among Catholic priests from four Spanish-speaking countries
Source: PLoS One. 2026 May 11;21(5):e0347608. doi: 10.1371/journal.pone.0347608 (PMC13160297; doi:10.1371/journal.pone.0347608)
Supplement: S1 Checklist — (DOCX) [file pone.0347608.s003.docx]

Inclusivity in global research

PLOS’ policy on inclusivity in global research aims to improve transparency in the reporting of research performed outside of researchers’ own country or community and ensures that PLOS publications reporting global research adhere to high standards for research ethics and authorship. Authors of relevant research articles may be asked to complete the questionnaire below, which outlines ethical, cultural, and scientific considerations specific to inclusivity in global research. This questionnaire may be requested when researchers have travelled to a different country to conduct research, if research uses samples collected in another country, research with Indigenous populations or their lands, or if research is on cultural artefacts. Researchers travelling to another country solely to use laboratory equipment will not normally be required to complete the questionnaire. However, the questionnaire can be requested at the journal’s discretion for any submission – if you have been requested to complete this questionnaire by the PLOS journal you submitted to, please do so.

Please complete the questionnaire below and include this as a Supporting Information file with your manuscript. Note that if your paper is accepted for publication, this checklist will be published with your article in the supporting information files. Please ensure that you reference the checklist in the main body of your manuscript. We suggest adding a subsection ‘Inclusivity in global research’ to your Methods section and adding the following sentence: “Additional information regarding the ethical, cultural, and scientific considerations specific to inclusivity in global research is included in the Supporting Information (SX Checklist)”

The questions have been designed to be applicable to a wide range of study types, and there are subsections for both human subjects research and non-human subjects research. If any of the questions are not relevant to your research please mark them as “N/A” as appropriate.

**Ethical considerations, permits and authorship**

*This section is applicable to all research types.*

Provide details as to who granted permissions and/or consent for the study to take place in the Methods section of your manuscript. This should include the names of **all** ethics boards, governmental organizations, community leaders or other bodies that provided approval for the study. If individuals provided approval refer to these people by their role or title but do not list their name(s).

Reported on page number: 14

If there were any deviations from the study protocol after approval was obtained please provide details of these changes in the Methods section of your manuscript.

Reported on page number: No deviations from the approved study protocol occurred during the conduct of the research.

Did this study involve local collaborators that are residents of the country where the research was conducted or members of the community studied? If you do not have any authors from said communities, please provide an explanation for this below.

Yes. The study included researchers based in Spain, where the research was conducted, ensuring familiarity with the local cultural and social context. Although some participants were from Latin American countries and no collaborators from these countries were directly involved in the authorship, all data collection was conducted remotely from Spain. Members of the research team also have prior extensive experience conducting research in Latin American contexts, as detailed in the Reflexivity and Positionality section. Additionally, co-authors based in the United States are leading contributors to the theoretical model underpinning this study, providing essential conceptual expertise.

Everyone listed as an author should meet PLOS’ criteria for authorship and all individuals who meet these criteria should be included in the author byline, rather than the acknowledgements. For further information please see the journal’s Authorship Policy.

**Human subjects research (e.g. health research, medical research, cross-cultural psychology)**

Did you obtain written informed consent from a representative of the local community or region before the research took place? How did you establish who speaks for the community? Details of written informed consent obtained from study participants should be reported separately in the Methods section of your manuscript.

Not applicable. The study did not involve research with a formally defined community requiring authorization from a community representative. Instead, participation was based on individual informed consent obtained directly from each participant, as detailed in the Methods section.

How did members of the local community provide input on the aims of the research investigation, its methodology, and its anticipated outcome(s)?

The study was not designed as a participatory research project involving a formally defined community. However, the research aims and methodology were informed by the research team’s prior experience in the field, as well as by pilot interviews that helped refine the interview guide and ensure contextual relevance. Care was taken to ensure that the research questions and procedures were appropriate to the sociocultural context of the participants. In the broader research project team in which this study is embedded, there were members from several Latin American countries or who had lived in those countries for many years. These team members also provided cultural feedback before the interviews were conducted.

When engaging with the local community, how did you ensure that the informed consent documents and other materials could be understood by local stakeholders?

All study materials, including the informed consent documents, were provided in Spanish, the native language of the participants. The study was also explained verbally prior to participation, allowing participants to ask questions and seek clarification when needed, ensuring full understanding of the research procedures.

Will the findings of the research be made available in an understandable format to stakeholders in the community where the study was conducted (e.g. via a presentation, summary report, copies of publications, etc.)? Please provide details of how this will be achieved.

Yes. The findings have already been disseminated through two public presentations in Spain: one in an academic setting and another open to non-academic audiences, which also included online access to facilitate participation from international communities. In addition, all participants were offered the possibility to contact the research team if they wished to receive information about the study results. The article will be published in open access format, ensuring broad availability, and will be shared directly with interested stakeholders upon request.

**Non-human subjects research using specimens/ animals collected as part of the study, or those housed in archival collections. Examples include archaeology, paleontology, botany and zoology.**

Did the permission you obtained from a local authority to perform the study include an agreement on access to outputs and benefit sharing? This may include procedures to enable fair distribution of the benefits and resources arising from the research performed. Please include any details of Prior Informed Consent and Benefit Sharing Agreements obtained. These may be required by field-specific regulations, for example the Convention on Biological Diversity (CBD) and the associated Nagoya Protocol.

N/A

If the material used in your study was imported, please A) provide the year it was imported and B) indicate whether permits were obtained to import/export the materials used, C) provide details of any permits obtained. If this information is not available, please indicate this.

N/A

If you used archival specimens, please state how the material used in your study was acquired by the institute it is held in and provide details of any permits obtained for the original excavations/ sample collection. If this information is not available, please indicate this.

N/A

How was the potential cultural significance of the materials collected in your study to local communities considered in your research design? Were Indigenous peoples and/or local researchers and institutions involved with archaeological excavations / collection of specimens? If so, please provide a description of their involvement.

N/A

If your manuscript includes photographs of human remains please indicate whether authors obtained permission from descendants or affiliated cultural communities to do so.

N/A
