# Supplementary material for: Mediating reconciliation with God: Exploring divine forgiveness experiences during confession among Catholic priests from four Spanish-speaking countries
Source: PLoS One. 2026 May 11;21(5):e0347608. doi: 10.1371/journal.pone.0347608 (PMC13160297; doi:10.1371/journal.pone.0347608)
Supplement: Attachment — Submitted filename: Response to Reviewers.docx [file pone.0347608.s005.docx]

**Response to the Editor’s and Reviewers’ Comments**

Note: A separate cover letter summarizes the changes made to the manuscript. In this document, we provide a detailed, point-by-point response to each comment raised by the editor and reviewers

**Journal Requirements**
When submitting your revision, we need you to address these additional requirements.
1. Please ensure that your manuscript meets PLOS ONE's style requirements, including those for file naming. The PLOS ONE style templates can be found at
[https://journals.plos.org/plosone/s/file?id=wjVg/PLOSOne_formatting_sample_main_body.pdf](https://track.editorialmanager.com/CL0/https:%2F%2Fjournals.plos.org%2Fplosone%2Fs%2Ffile%3Fid=wjVg%2FPLOSOne_formatting_sample_main_body.pdf/1/010f019d039e840d-063c18b5-ef78-458e-bb2c-4c2653498f77-000000/4z8QhEJhXKyFjzHcHvUQFHrFmPE3HdXALxA03g1qFHo=255) and [https://journals.plos.org/plosone/s/file?id=ba62/PLOSOne_formatting_sample_title_authors_affiliations.pdf](https://track.editorialmanager.com/CL0/https:%2F%2Fjournals.plos.org%2Fplosone%2Fs%2Ffile%3Fid=ba62%2FPLOSOne_formatting_sample_title_authors_affiliations.pdf/1/010f019d039e840d-063c18b5-ef78-458e-bb2c-4c2653498f77-000000/iuBU-i2NylvrmgtdgxQ0wQAaYIP4ulsQh74nJq4myOs=255)

**Response to Journal Requirement #1**: We thank the editor for this request. We have carefully adhered to all PLOS ONE formatting requirements and ensured that the manuscript fully complies with the journal’s style guidelines, including those related to file naming.

2. Please include a complete copy of PLOS’ questionnaire on inclusivity in global research in your revised manuscript. Our policy for research in this area aims to improve transparency in the reporting of research performed outside of researchers’ own country or community. The policy applies to researchers who have travelled to a different country to conduct research, research with Indigenous populations or their lands, and research on cultural artefacts. The questionnaire can also be requested at the journal’s discretion for any other submissions, even if these conditions are not met. Please find more information on the policy and a link to download a blank copy of the questionnaire here: <https://journals.plos.org/plosone/s/best-practices-in-research-reporting>. Please upload a completed version of your questionnaire as Supporting Information when you resubmit your manuscript.

**Response to Journal Requirement #2**: The PLOS inclusivity in the global research questionnaire has been completed and uploaded as Supporting Information (S3 Checklist) with the revised submission.

3. Your ethics statement should only appear in the Methods section of your manuscript. If your ethics statement is written in any section besides the Methods, please delete it from any other section.

**Response to Journal Requirement #3**: We have checked that the ethics statement appears exclusively in the Methods section and does not appear elsewhere in the manuscript.

4. When completing the data availability statement of the submission form, you indicated that you will make your data available on acceptance. We strongly recommend all authors decide on a data sharing plan before acceptance, as the process can be lengthy and hold up publication timelines. Please note that, though access restrictions are acceptable now, your entire data will need to be made freely accessible if your manuscript is accepted for publication. This policy applies to all data except where public deposition would breach compliance with the protocol approved by your research ethics board. If you are unable to adhere to our open data policy, please kindly revise your statement to explain your reasoning and we will seek the editor's input on an exemption. Please be assured that, once you have provided your new statement, the assessment of your exemption will not hold up the peer review process.

**Response to Journal Requirement #4:** We thank the editor for this clarification. In line with the journal’s data availability policy, the data have now been made publicly accessible, as specified in the Data Availability Statement.

5. Please amend your authorship list in your manuscript file to include author Martiño Rodríguez-González, Carmen Callizo, Maria Calatrava, Maria Pilar Martínez-Díaz, Maria Pilar Martínez-Díaz, Richard G. Cowden.

6. Please amend the manuscript submission data (via Edit Submission) to include author Rodríguez-González, M., Callizo, C., Calatrava, M., Martínez Díaz, M.ª P., Cook, K., & Cowden, R. G.

**Response to Journal Requirement #5&6**: We thank the editor for this comment. However, we are unclear about this request, as we have verified that the authors listed in the submission system are consistent with those and their order in the manuscript. We would be grateful for further clarification. We confirm that the authors of the manuscript and the order they should be listed is as follows: Callizo, C., Rodríguez-González, M., Calatrava, M., Martínez-Díaz, M.ª P., Cook, K., & Cowden, R. G.

7. Your abstract cannot contain citations. Please only include citations in the body text of the manuscript, and ensure that they remain in ascending numerical order on first mention.

**Response to Journal Requirement #7:** We confirm that the abstract does not contain any citations and complies with the journal’s guidelines.

8. If the reviewer comments include a recommendation to cite specific previously published works, please review and evaluate these publications to determine whether they are relevant and should be cited. There is no requirement to cite these works unless the editor has indicated otherwise.

**Response to Journal Requirement #8:** We have carefully evaluated the references suggested by the reviewers. Following this assessment, we have included two of them, as they are relevant to our work, as detailed in our responses to Reviewer #2.

9. Please review your reference list to ensure that it is complete and correct. If you have cited papers that have been retracted, please include the rationale for doing so in the manuscript text, or remove these references and replace them with relevant current references. Any changes to the reference list should be mentioned in the rebuttal letter that accompanies your revised manuscript. If you need to cite a retracted article, indicate the article’s retracted status in the References list and also include a citation and full reference for the retraction notice.

**Response to Journal Requirement #9:** We have carefully reviewed and updated the reference list to ensure its completeness and accuracy. Specifically, we have added two references recommended by the reviewer (now References 18 and 40), as well as an additional relevant reference (Reference 46). We have also updated a previously “in press” article to its final published version (Reference 15). No previously included references have been removed. In addition, the entire reference list has been formatted according to the Vancouver style required by PLOS ONE.

[Note: HTML markup is below. Please do not edit.]

**Reviewers' comments:**
Reviewer's Responses to Questions

**Comments to the Author**

1. Is the manuscript technically sound, and do the data support the conclusions?

The manuscript must describe a technically sound piece of scientific research with data that supports the conclusions. Experiments must have been conducted rigorously, with appropriate controls, replication, and sample sizes. The conclusions must be drawn appropriately based on the data presented.

Reviewer #1: Yes

Reviewer #2: Yes

2. Has the statistical analysis been performed appropriately and rigorously?

Reviewer #1: N/A

Reviewer #2: N/A

3. Have the authors made all data underlying the findings in their manuscript fully available?

The [PLOS Data policy](http://track.editorialmanager.com/CL0/http:%2F%2Fwww.plosone.org%2Fstatic%2Fpolicies.action%23sharing/1/010f019d039e840d-063c18b5-ef78-458e-bb2c-4c2653498f77-000000/0lgkZO5wOe4prRNoLM0Skl_Whnm0vXMeiV5Igo8NEPA=255) requires authors to make all data underlying the findings described in their manuscript fully available without restriction, with rare exception (please refer to the Data Availability Statement in the manuscript PDF file). The data should be provided as part of the manuscript or its supporting information, or deposited to a public repository. For example, in addition to summary statistics, the data points behind means, medians and variance measures should be available. If there are restrictions on publicly sharing data—e.g. participant privacy or use of data from a third party—those must be specified.

Reviewer #1: Yes

Reviewer #2: Yes

4. Is the manuscript presented in an intelligible fashion and written in standard English?

PLOS ONE does not copyedit accepted manuscripts, so the language in submitted articles must be clear, correct, and unambiguous. Any typographical or grammatical errors should be corrected at revision, so please note any specific errors here.

Reviewer #1: Yes

Reviewer #2: Yes

5. Review Comments to the Author

Please use the space provided to explain your answers to the questions above. You may also include additional comments for the author, including concerns about dual publication, research ethics, or publication ethics. (Please upload your review as an attachment if it exceeds 20,000 characters)

**Reviewer #1**

**R1#C#0:** This manuscript presents a carefully conducted qualitative study exploring Catholic priests’ experiences and understandings of divine forgiveness (DF) within sacramental confession. The topic is original, theoretically engaged, and timely within the emerging psychological literature on divine forgiveness.

One of the strongest aspects of the manuscript is the rigorous and sustained engagement with the two central theoretical models (Fincham & May; Cook & Cowden). The authors not only present these models clearly and accurately in the Introduction, but also maintain them as analytic reference points throughout the Results and Discussion. The models are neither superficially cited nor merely decorative; rather, they structure the analytic logic of the study and are revisited critically in light of the data. This theoretical coherence significantly strengthens the manuscript and distinguishes it from purely descriptive qualitative work.

The study is methodologically transparent, the analytic process is clearly described, and the integration with existing theoretical models is thoughtful. The manuscript has clear potential for publication. However, several conceptual and interpretive issues require clarification before it can be considered suitable for publication in a general scientific journal such as PLOS ONE.

**R1#C#0_Response:** We thank the reviewer for their careful reading of the manuscript and for their thoughtful and constructive comments. We particularly appreciate the positive evaluation of the study’s originality, methodological transparency, and theoretical engagement, as well as the recognition of our sustained and rigorous use of the two central theoretical models throughout the manuscript.

We also greatly value the reviewer’s suggestions for improvement. We have carefully considered all the points raised and have revised the manuscript accordingly. Below, we respond to each comment in detail and indicate how the manuscript has been revised in response.

My comments focus on strengthening theoretical precision, interpretive restraint, and epistemological clarity.

**R1#C#1:** 1. Scope of Theoretical Claims

The manuscript proposes that the confessor may constitute a distinct “fourth vertex” in the relational spirituality model of personal sin. While this is a compelling interpretive insight, the current formulation risks overstating the implications of findings derived from a small and relatively homogeneous sample (N = 10 experienced male priests, predominantly from Spain).

I recommend moderating the strength of this claim. Rather than suggesting structural modification of the model, the argument would be more appropriately framed as a context-specific elaboration within sacramental Catholic settings, and a hypothesis-generating extension requiring further empirical examination. The contribution remains valuable, but the language should reflect its exploratory status.

**R1#C#1_Response:** We thank the reviewer for this helpful comment and agree that the original formulation may have overstated the theoretical implications of our findings.

In response, we have revised several passages in the Discussion to moderate the strength of our claims and more clearly reflect their exploratory and context-specific nature. Specifically, we now frame our interpretations as emerging from “this sample and context,” adopt more tentative language throughout (e.g., “appears to,” “may be experienced as”), and clarify that the role of the confessor is described from participants’ perspectives rather than as a generalizable structural feature. Importantly, the passage proposing the “fourth vertex” has been reformulated to emphasize that this is a tentative, hypothesis-generating extension, and we explicitly state that it should not be understood as a structural modification of the model but as a contextually grounded elaboration within sacramental Catholic settings (see revised Discussion section).

We have also aligned Fig 2 and its caption with this more cautious framing. The figure is now described as a contextually grounded, exploratory adaptation based on a small sample, and the confessor is presented as being provisionally conceptualized as a possible additional relational element. The description of the relational links has also been revised using more tentative language (see Fig 2’s caption). We hope these revisions ensure that the scope of our theoretical claims is appropriately aligned with the exploratory nature of the study.

**R1#C#2:** 2. Distinction Between Psychological Description and Theological Normativity

The manuscript generally maintains a psychological lens; however, in several passages—particularly in the Results and Discussion—the wording occasionally moves very close to doctrinal formulation rather than analytic description.

For example: In the Results, statements such as “You can only be certain of God’s forgiveness when the priest… says ‘I absolve you’” are presented with minimal analytic framing. While clearly derived from participant quotations, the surrounding text could more explicitly indicate that this reflects a participant belief structure rather than an ontological claim about the nature of forgiveness. In the Discussion, the description of the confessor as one who “sacramentally identifies with Christ and enacts DF” reads, at points, as a theological assertion rather than as an account of how priests conceptualize their role. A small shift in phrasing (e.g., “is understood by participants as…”) would maintain scientific neutrality. Similarly, when describing absolution as the moment in which the penitent “is reunited with God,” the manuscript could clarify that this is a phenomenological or doctrinally mediated interpretation rather than an empirically demonstrable event.

None of these instances undermine the study. However, because PLOS ONE is a general scientific journal, maintaining a consistently descriptive psychological register—especially when dealing with sacramental theology—would enhance epistemological clarity. The suggested revisions require only slight adjustments in phrasing, not substantive reanalysis.

**R1#C#2_Response:** We thank the reviewer for this thoughtful comment and agree that, in several passages, the wording could be read as moving too close to doctrinal formulation rather than remaining consistently within a descriptive psychological register.

In response, we have carefully revised the relevant passages in both the Results and Discussion to clarify that these statements refer to participants’ understandings, experiences, and belief structures, rather than to ontological claims about the nature of divine forgiveness. Specifically, we introduced more descriptive phrasing throughout (e.g., “was described by participants as,” “is understood within Catholic doctrine, and by participants, as,” “may be experienced as,” “appears in participants’ accounts as”) and adjusted several formulations that could have sounded overly theological or normative. We also revised instances referring to reconciliation with God and absolution to make clearer that these are phenomenological or doctrinally mediated interpretations rather than empirically demonstrable claims. Please see the revised manuscript with tracked changes/marked changes for the exact wording modifications in the Results and Discussion sections.

**R1#C#3:** 3. Cultural Framing and Representativeness

Although the study includes participants from four Spanish-speaking countries, six of the ten participants are based in Spain. At several points, the manuscript refers broadly to “Spanish-speaking contexts.” I recommend slightly narrowing this phrasing or explicitly acknowledging the uneven distribution of participants across countries to avoid unintended generalization.

**R1#C#3_Response:** Thank you for this helpful observation. We agree that the distribution of participants across countries warrants a more cautious cultural framing. In response, we have revised the manuscript to avoid broad generalizations to “Spanish-speaking contexts” and instead use more qualified language (e.g., specifying when findings reflect this sample). We have also incorporated a clarification in the Limitations section noting that most participants were based in Spain, which may have influenced the scope of the findings.

**R1#C#4:** 4. Use of AI as Analytic Aid

The transparency regarding the use of ChatGPT as an auxiliary analytic tool is commendable. Given the novelty of AI-assisted qualitative analysis, I suggest slightly expanding the clarification that: AI did not generate primary codes or interpretive structures, all analytic decisions remained with the research team, and the tool was used solely for robustness checks.
This would pre-empt potential methodological concerns.

**R1#C#4_Response:** Thank you for this helpful suggestion. We have reformulated the corresponding section in the Methods to clarify the role of ChatGPT in the analytic process. Specifically, we now explicitly state that the AI tool did not generate primary codes or interpretive structures, that all analytic decisions remained under the full control of the research team, and that it was used exclusively for robustness checks.

**R1#C#5:** Overall Evaluation

This is a conceptually strong, theoretically coherent, and methodologically careful qualitative study that meaningfully advances empirical research on divine forgiveness within a sacramental Catholic context. The sustained integration of contemporary theoretical models is particularly noteworthy.

With modest refinements to theoretical framing and epistemological positioning, the manuscript would represent a valuable contribution to the field.

I encourage revision along the lines suggested above.

**R1#C#6_Response:** Thank you for this generous and encouraging evaluation of our work. We have carefully revised the manuscript as shown above. We believe these changes have strengthened the clarity and coherence of the manuscript, and we are grateful for your constructive guidance in this process!

**Reviewer #2**

Review of Manuscript PONE-D-25-62289: "Mediating Reconciliation With God: Exploring Divine Forgiveness Experiences During Confession Among Catholic Priests From Four Spanish-Speaking Countries"

**R2#C#0:** Dear Authors:

Thank you for the opportunity the read and review your article entitled "Mediating Reconciliation With God: Exploring Divine Forgiveness Experiences During Confession Among Catholic Priests From Four Spanish-Speaking Countries." I begin by briefly noting some pronounced strengths of the manuscript.

Strengths

1 – The focus on reports of Catholic priests as confessors is quite novel and innovative. I am not Catholic but the authors provide a deep but “outsider friendly” view of the focal phenomenon. I found the piece deeply engaging.

2 – The Referencing and citations were nearly perfect. The manuscript was lucid and clear … and virtually error free. The piece was well-cited, with heavy reliance on very fresh and recent sources (many from the past 5 years or so).

3 – In terms of qualitative methodology, the piece was rigorous and systematic. The depth of the interviews (mean = 74 minutes) is rare these days. I was also impressed with the use of ChatGPT as an “auxiliary aid” (or audit tool).


In the spirit of precision and constructive criticism, I offer the following recommendations that I hope will be helpful.

**R2#C#0_Response:** Thank you very much for your thoughtful and encouraging evaluation of our manuscript. We sincerely appreciate your recognition of the study’s novelty, methodological rigor, and clarity, as well as your positive assessment of the theoretical integration and use of recent literature.

We are also grateful for the constructive recommendations provided below. These comments have been helpful in further strengthening the manuscript. In response, we have carefully revised the text accordingly, and below we detail how each point has been addressed.

**R2#C#1:** 1 – On p. 4, line 71, you reference “relational spirituality.” I encourage you to cite Annette Mahoney here (in addition to those already cited). She, I believe, was the innovator of this concept.

**R2#C#1_Response:** We agree that Annette Mahoney’s work has been foundational in the development of the concept of relational spirituality. Accordingly, we have added a citation to Mahoney (2010) at the first mention of this framework in the discussion.

**R2#C#2:** 2 – Page 7, line 149. In APA 7th Style, when multiple references are cited parenthetically at the end of a statement, these references need to alphabetized by the first authors’ last names. For example, when you make a statement and then cite (Ramirez, 1985; Ruiz, 2005; Gomez, 2003) then Gomez needs to go before Ramirez.

**R2#C#2_Response:** Thank you for this observation. We acknowledge that there was one inconsistency in the author order, which has now been corrected following APA guidelines. In the final version of the manuscript, the references have been reformatted according to the Vancouver style required by the journal, and the citation order has been adjusted to reflect the order of appearance in the text.

**R2#C#3:** 3 – An uncited piece you may want to consider in connection with Confession is:
White, T., et al. (2018). Kept together by faith: Confession and forgiveness among American Catholic and Orthodox Christian families. Marriage & Family Review, 54, 677-692. doi:10.1080/01494929.2018.1469573

**R2#C#3_Response:** We agree that White et al. (2018) provides a valuable perspective on the relational and communal dimensions of confession and forgiveness in Catholic contexts. Accordingly, we have incorporated this reference in the Introduction when describing confession as a relationally mediated sacramental practice.

**R2#C#4:** 4 – On p. 10, you mention a sample of “ten Catholic priests” (line 203), but the numbers given on lines 214-215: “Spain – 6, Peru – 1, Chile – 1, and Mexico – 1” only total 9. Please correct this inconsistency.

**R2#C#4_Response:** Thank you for noting this inconsistency. This has now been corrected. The sample consists of ten Catholic priests; we had inadvertently omitted one Spanish participant in the country breakdown, which has now been included.

**R2#C#5:** 5 - Please be careful about “naked” paragraphs—meaning paragraphs without a single reference to the scholarly literature. Occasionally, there may be a need for such a paragraph but I would typically avoid going for too long without anchoring and re-anchoring your work in the scholarly literature. Some “naked” paragraphs occur on pages 13-14 (only one cite on these two pages), and for most of p. 32.

**R2#C#5_Response:** Thank you for this observation. Regarding (former) pages 13–14, these sections correspond to the description of participants. As such, they are primarily descriptive and based on the study data, so no additional references were deemed necessary in those paragraphs. Regarding page 32, we believe there may have been some confusion, as part of the text corresponds to a figure caption that may visually resemble a paragraph. We have carefully rechecked the Discussion section to confirm that all interpretative paragraphs are appropriately anchored in the scholarly literature, and two references have been added (see responses to comments R2#C#1, R2#C#3).

**R2#C#6:** 6 – Typically, in qualitative work, the “Results” section is instead labeled as “Findings.” (p. 15)

**R2#C#6_Response:** Thank you for this suggestion. We agree that “Findings” is commonly used in qualitative research, and we have now revised the manuscript accordingly: The section previously labeled Results has been changed to Findings.

**R2#C#7:** 7 – In the Results/Findings section, I would recommend listing/outlining your major Themes up front (after the first paragraph on p. 15), in the spirit of “tell the reader what you are going to tell them.” Additionally, on a related note, I recommend similarly outlining the sub-themes at the beginning of each theme. Both strategies serve as a reader guide and orientation.

**R2#C#7_Response:** Thank you for this helpful suggestion. We have revised the Findings section to improve clarity and reader orientation. Specifically, we now introduce the main themes at the beginning of the section. While we have not added the sub-themes at the beginning of each subsection in order to preserve the narrative flow, we have included a reference to Supplementary Table S2, which provides a structured overview of all themes and sub-themes. This allows readers to easily follow the organization of the analysis while maintaining readability within each theme.

**R2#C#8:** 8 – I found the narrative selections to be brief but excellent. I also appreciated the decision to italicize the Priests’ quotes for differentiation and clarity. However, on p. 21 (lines 470-471) you forgot to italicize a priest’s quote, I believe.

**R2#C#8_Response:** Thank you for noting this. This has now been corrected.

**R2#C#9**
9 – One slightly jarring feature of the piece is a lack of a transition sentence or two between the major Themes of the Results/Findings section. I recommend adding transitions between Themes 1 and 2, 2 and 3 (p.23), etc. Please help the reader see the connection between the Themes and why you ordered the presentation of the Themes as you did.

**R2#C#9_Response:** Thank you for this insightful suggestion. We agree that the flow between themes can be further clarified to improve readability and to make the analytical structure more explicit. In response, we have added brief transition paragraphs at the end of each theme in the Results/Findings section. These transitions are intended to (a) summarize the key insight of the preceding theme, (b) highlight its conceptual link with the subsequent theme, and (c) make explicit the rationale for the ordering of themes. The following transition sentences have been incorporated into the revised manuscript:

Between Theme 1 and Theme 2:

“Taken together, these accounts highlight confession as a fundamentally relational and lived experience, embedded in ongoing interpersonal dynamics. Building on this relational foundation, the following theme explores how these experiences unfold as dynamic processes of reconciliation over time.”

Between Theme 2 and Theme 3:

“While these dynamic movements of reconciliation illustrate how the process ideally unfolds, participants also pointed to conditions that can hinder or facilitate these trajectories. The next theme therefore examines the perceived barriers and facilitators shaping these experiences.”

Between Theme 3 and Theme 4:

“These barriers and facilitators not only structure the process externally but are also deeply intertwined with participants’ internal experiences. To better understand this subjective dimension, the next theme focuses on the cognitive and emotional aspects of DF.”

Between Theme 4 and Theme 5:

“Taken together, the cognitive and emotional dimensions reveal how participants make sense of and engage with DF at an individual level. The final theme integrates these insights with the relational and dynamic aspects previously described, offering a more comprehensive understanding of the phenomenon.”

We have also included an introductory sentence added at the beginning of Theme 5:

“Bringing together the relational, processual, and experiential dimensions identified in the previous themes, this integrative axis highlights how these elements converge into a coherent understanding of DF.”

**R2#C#10:** 10 – Page 36, line 810, needs to be indented.

**R2#C#10_Response:** Thank you for noting this formatting issue. The paragraph has now been correctly indented!

**R2#C#11**
I wish you the best as you continue your efforts to publish this manuscript, it is an excellent piece and was a pleasure to read.

**R2#C#11_Response:** We sincerely thank the reviewer for their kind and encouraging words and appreciate their thoughtful engagement with our work.
